# Challenges of establishing a road traffic injury surveillance system: a qualitative study in a middle-income country

**DOI:** 10.5249/jivr.v11i2.1228

**Published:** 2019-07

**Authors:** Sakineh Sharifian, Reza Khani Jazani, Homayoun Sadeghi-Bazargani, Davoud Khorasani-Zavareh

**Affiliations:** ^*a*^Department of Health in Emergencies and Disasters, School of Public Health and Safety, Shahid Beheshti University of Medical Sciences, Tehran, Iran.; ^*b*^Road Traffic Injury Research Center, Department of Statistics and Epidemiology, Tabriz University of Medical Sciences, Tabriz, Iran.; ^*c*^Department of Neurobiology, Care Sciences and Society, Division of Family Medicine and Primary Care, Karolinska Institutet, Stockholm, Sweden.

**Keywords:** Qualitative study, Surveillance, Road traffic injury, Crash, Stakeholder

## Abstract

**Background:**

Establishing effective road traffic injury surveillance is an important priority for low- and middle-income countries since a large proportion of fatal road traffic injuries occur in these countries. A surveillance system can coordinate the activities and compile the data gathered by all road safety organizations. This study aims to explore the challenges of establishing road traffic injury surveillance based on the stakeholders’ experiences.

**Methods:**

This is a qualitative content analysis study that was performed in 2018 in Iran. The study was conducted through interviews with 13 participants and employed purposeful sampling. Data generation was continued until concept saturation.

**Results:**

Five main categories and 17 sub-categories were identified including: policy-making (legal authority, stakeholders, content policy and plan); process (collection and recording, analysis and interpretation, dissemination and feedback); resources and infrastructure (technology, staff, structure, finance); coherence and coordination (communication, dispersion, cooperation, consensus); and context (socio-cultural, political).

**Conclusions:**

By creating a clear structure for a road traffic injury surveillance system, identifying data collection systems and stakeholders in the field of road safety and injury prevention, determining a clear goal for improving road safety, and formulating policies for the dissemination of road traffic crash data among stakeholders, it will be possible to overcome the obstacles to establishing a surveillance system for road traffic injury.

## Introduction

Road traffic injuries (RTIs) are a major issue in the world, especially in low- and middle-income countries (LMICs).^1,2^ In Iran, RTIs are the second major cause of death according to the World Health Organization (WHO) report and the burden of road traffic injuries derives from accidents involving motor vehicles and predominantly affected economically productive age groups.^3^ Establishment of injury surveillance is an important step for a better understanding of the problem thus leasing to RTI prevention.^4^ However, one of the main problems with designing and implementing fatal and non-fatal road traffic injury prevention strategies is the lack of actual and qualified related data.^5-7^ The literature has emphasized the importance of precise and reliable data in influencing road safety policy and any preventive activities.^8-10^ Along with collecting qualified data; analysis, interpretation, and dissemination of health information and getting feedback to the beneficiaries must be considered. ^11^

Data registration and establishing a surveillance systemfor road traffic injury is an important concern in most LMICs, because the burden of RTIs is high in these countries and the interventions are often not based on the available evidence.^7,12,13^ WHO defines surveillance as “the continuous, systematic collection, analysis and interpretation of health-related data needed for the planning, implementation, and evaluation of public health practice”.^14^ In LMICs, road traffic data, often collected from different sources;^15^ usually suffers from under-reporting.^16^ High-income countries, usually have well-established surveillance systems , while most LMICs, don’t have trauma registry and surveillance systems at all or have incomplete ones.^17^ These systems may have some problems such as lack of precise data for pre- and post-hospital death, deficiency in injury data registry in the hospital especially for outpatients, and ambiguity in the definition of variables in injury registry. ^18-20^ There is a clear need for a national road traffic injury surveillance to coordinate all organizations’ activities.^21,22^

Injury surveillance has the potential to identify the trends, the populations at risk, injury mechanism and the risk factors in order to design and evaluate priorities in injury prevention and evaluate the effectiveness of interventions.^14^ Due to the importance of this system, there are few examples of establishing a road traffic surveillance system,^12,13^ which suffers from under reporting. In recent years, a few epidemiological studies have been conducted in Iran on the injury surveillance system.^18,23-25^ Based on the literature, in Iran data gathering and registry systems for RTIs are passive, so, many data, especially from rural areas, may be missed.^18,22,23,26^ In order to have a deeper understanding of the challenges of the surveillance system, it is necessary to have a deeper understanding of the system based on stakeholders’ experiences.^27^ Only through a deep qualitative study, can we understand the experiences of those who have interacted with the process of recording data and explain the challenges of the process of establishing a surveillance system. This study, then, was designed to explore the barriers to establishing an integrated passive road traffic injury surveillance system in Iran according to the experiences of involved stakeholders.

## Materials and Methods 

This qualitative study was performed at national level during July 2017 to March 2018.The qualitative content analysis approach by Graneheim and Lundman^28^ was used to explain the challenges of establishing a surveillance system.

**Participants’ selection**

The participants were 13 individuals from different authorities such as the Emergency Department, the Police, the Ministry of Health and (MOHME), and faculty members. The project researchers and the staff involved in the process of recording, analysis, interpretation, and dissemination of data had experience or academic education in the field of traffic or injury prevention and safety promotion as well as publishing papers or books regarding to this study. Participants were chosen using the purposive sampling at different organizational levels. The exclusion criteria were lack of interest in the interview or being incapable of sharing their experiences.

**Data generation**

Initially, three non-structured interviews were conducted. Primary questions were designed based on the opinions of the research team and the concepts obtained from the interviews. Afterwards, ten semi-structured interviews were performed in order to collect the data. An interview guide was used to explore the barriers to establishing a surveillance system. Questions were covered, such as: What is your personal experience about road traffic injury surveillance? In your experience, what are the problems and solutions for establishing a national traffic surveillance system? And based on your experience, what are the desired characteristics of an effective registry system in Iran?

To conduct the interview, the participants were first contacted directly by the principle investigator or by a phone call. Then, the interview was performed by (SSH) in Persian. The interviews were recorded with the participants’permission. Moreover, during the interview, the note-taking technique was used in order to design questions for the next interviews. Data collection was continued until question and concept saturation occurred.^29^ Interview times varied from 30 to 60 minutes. Immediately after the end of each interview, the audio files were transcribed verbatim and then written down using Word Office 2007.

**Data analysis**

Each interview was listened to several times. After transcribing verbatim, texts were again compared to the audio files and then the first four transcribed were analyzed by both the principle investigator and the supervisor until consensus was reached on data processing. To analyze the data, the five-step data analysis approach was used. ^28^ In the first step, key concepts were determined by means of transcripts, line-by-line. Then a conceptual framework was designed based on the key concepts; and other data were extracted and structured based on this framework. Afterwards, the data were categorized into different main categories and sub-categories, based on the relationship between codes. All the steps were performed first in Persian and then they were translated into English.

**Rigor**

Based on the Trustworthiness criteria of Lincoln and Gub^30^ four criteria including confirmability, credibility, dependability, and transferability were used to evaluate the quality of results. To ensure credibility, adequate time was used to collect the data and we strived to be accurate at all the steps of data generation and analysis. To ensure collection of the required documentation, we tried to use participants who have the most experience and understanding on the subject of the study. In order to determine the consistency of the understanding of the generated data, they were checked by a PhD student as external auditors (as investigator triangulation and peer check) and the research supervisor, DKZ (expert check) who is an expert in qualitative research, safety promotion and prevention of injury. The transcribed texts of the interviews were given to the participants to get their corrective comments (Member check). Moreover, the main researchers were SSH, Student of Disaster Health and Emergencies and DKZ who has been active in this area for years; both had experience of working in this field. The research team was comprised of four experts in qualitative studies and the road crash recording systems and all were extremely familiar with the road safety context. For dependability, the study method was fully described in the method section and the study process was recorded in all steps in order to reach auditability. Confirmability was provided by reflectivity and investigator triangulation. In order to increase the scientific accuracy during the study, bracketing process was used to prevent the researchers’ subjective opinions from affecting the research. The process of study is fully described in this manuscript in order to ensure transferability.

## Results

At first, 800 primary codes were extracted, of which after constant comparisons, turned into 350 final codes in 17 sub-categories and 5 main categories. These were: Policy-making (legal authority, stakeholders, content policy and plan); Process (collection and recording, analysis and interpretation, dissemination and feedback); Resources and Infrastructure (technology, human, structural, financial), Coherence and Coordination (communication, dispersion, cooperation, consensus) and Context (Scio- cultural, political)(Table 1).

**Table 1:** The challenges of establishing a road traffic injury surveillance system in Iran.

Category	Sub-category	Code samples
**Policy-making**	Legal authority	Indeterminate custodianship of the surveillance system
		Lack of legislation rights for the governing organization
	Stakeholders	Lack of ability to identify stakeholders
		Poor use of the experts’ opinions
		Lack of trust between the stakeholders
	Policy content	Poor surveillance policy
		Weak monitoring and evaluation policies
	Plan	Visceral decision in prioritizing plans
		Failure to execute and follow up the plans
**Process**	Collection and recording	Multiple forms
		Lack of registration concern
	Analysis and interpretation	Lack of consensus among the experts
		Lack of efficiency of the interpretations for policy making
	Dissemination and feedback	Censoring the information
		Perceiving the data as a source of power
**Resources and Infrastructure**	Technology	Improper software
		Poor technological infrastructures
	Human	Multi-responsibility staff
		Lack of dedicated registrar
	Structural	Administrative and organizational instability
		Parallel structures in the concerned organization
	Financial	Lack of finance for registering
		High costs of establishing a surveillance system
**Coherence and Coordination**	Communication	Poor internal and external communication between organizations
		Failure in data linkage
	Dispersion	Parallel plans in organizations
		Isolated registration
	Cooperation	Poor inter-organizational cooperation
		Lack of memorandum of understandings (MOUs)
	Consensus	Lack of consensus among stakeholders in the surveillance process
		Lack of a unified definition
**Context**	Scio- cultural	Poor teamwork culture
		The blaming approach
	Political	Political interference in prioritizing plans
		Perceiving information as confidential

**Policy-making**

Legal authority

According to the participant viewpoints, lack of custodianship in road safety is a challenge in establishing road traffic injury surveillance. This is because the organizations in this field all perform their measures in a parallel and sporadic manner. Another point is the necessity of legislation rights for the legal agency of road safety to pass and enforce effective interventions.

(P13) … it doesn’t matter who takes the responsibility? The most important thing is that there is no supervision to coordinate the current organizations…. (P04) …. Prevention programs are executed in organizations in a parallel manner…

Stakeholders

Based on the experiences of the participants, weakness in recognizing the stakeholders, the lack of systematic communication and the absence of trust between organizations are challenges related to stakeholders. There is no clear regulation to define the communication between organizations. Lack of trust among stakeholders is the other challenge that leads to the duplication of work in collecting road traffic injury data and has a negative impact on their data exchange.

 (P 07) nobody trusts anybody else’s work, because they think that their own work is methodologically right and the others’ work is wrong… (P13) … Communication between the organizations must legally be included in their job descriptions…

Policycontent

Based on participants’ opinions, policies regarding road safety are very vague. Clear rules or guidelines should be compiled regarding data recording and dissemination, so that each organization does not make independent decisions about the registering and dissemination of data. Moreover, regulation on system evaluation in the organization must be developed.

(P05) … there is no policy for evaluating the existing systems in our country… (P03) … unfortunately one of the main reasons is that in our country, policies are made subjectively…

Plan

Based on the participants’ experience, road safety plans are mostly based on the viewpoints of the authorities rather than valid evidence. Moreover, most of the plans are formulated based on the mortality indicators while the crash injury and disability indicators are ignored. Another challenge is the lack of joint plans among the stakeholders.

 (P 09) … Why has all of the mortality caused by road traffic crashes suddenly become important for us? Because the world is focused on the mortality caused by road traffic crashes, so we focus on that too. We don’t have a coherent system for injuries.

**Process**

Collectionand recording

Overall, lack of data coverage, poor data collection system, and poor determination regarding a minimum dataset are some of the challenges related to this subcategory. Most of participants emphasized that the importance of recording must be understood by the stakeholders. Many of the core variables are unclear in recording forms and the main cause of death or injury is not mentioned. Low validity and reliability of the instruments is another challenge.

 (P 05) … In approximately 10 to 15% of the crash data recorded, we observe under-reporting... (P01) ... There are instruments that need to be corrected. The structural validity of the tools must be assessed...

Another important challenge is failure in recording the minimum dataset for the road traffic injury surveillance system. Some of data related to the disability caused by crashes as well as social factors affecting health, such as drug and alcohol abuse, should be collected and recorded by the organizations. Timeliness of information is also one of the challenges. At present, data are not interpreted on time and do not have much utility.

 (P10) … some of the necessary data related to crashes are not recorded, for example the outcome of the injury … (P03) … information is not provided to us in time to formulate interventions…

Analysis and interpretation

Most of participants commented on the low efficiency of data for crash data analysis and interpretation. Consequently, the data lacks adequate efficiency for making decisions, because the analyses are done merely to provide a report. Also, the analyzed data is not interpreted correctly by the experts. Another challenge is the lack of evidence-based studies based on the information.

 (P13) … defective recorded data by different organizations leads to an incorrect final analysis. You can see in WHO reports that they report two to three thousand deaths more than the announced death rate (by Iran) … (P10) …our biggest weakness is in data analysis and dissemination…

Another challenge is the existence of nothing more than a registration system in organizations. They collect and record data, however only in some cases do they analyze them, and then very superficially.

 (P07) … we don’t know the burden of road traffic injuries in the country, so, we encounter problems in prioritizing … (P06) …The main problem in (data) analysis is that in order to show good results, we change our criteria each year; in such a way that they are not comparable to the previous results...

Dissemination and feedback

According to the participants’ viewpoints, one of the challenges is the perception of data as something confidential. So, the organizations refrain from distributing it for research purposes and only consider their own interests when distributing the data. Data is only reported to the higher authorities, so data exchange is not done among stakeholders or to the lower levels. Also, the general public, the most important beneficiary, receives little information in this field. Another challenge is the fear of diminished authority due to distributing the data. Each organization’s information is considered as its personal asset. There is a dread among organizations about data dissemination: they will lose their data ownership, and then find other stakeholders using it without disclosing its source. Also, some of participants believed that by distributing the information, the power of the organization will diminish. Thus, conducting effective research in the field of road safety is not easy.

 (P09) … honestly, data dissemination is awful since we think that our data is confidential… (P03) … the problem is that every organization thinks that its data is its power and in practice it is…

**Resources and infrastructure**

Technological resources

One of the challenges in this category is the weakness of the technological infrastructures. In most regions in Iran, the automation infrastructures do not exist. Moreover, data collection systems do not have enough capacity to satisfy the stakeholders. The problem of linking data between organizations is another challenge.

 (P 13) … lack of linking among the organizations is partly due to the fact that our infrastructure is not built for it… (P 04) …the software doesn't have any support and needs to be improved…

Human resources

Overall, the lack of qualified staff and the heavy workload are the main challenges in the category of human resources. Staff working in data collection systems has multiple responsibilities. Therefore, due to their heavy workload, they do not pay enough attention to data collection and recording. Lack of a dedicated registrar was also a challenge. The registrar competency should be determined, but unfortunately this issue is neglected and the essential training is not taken seriously.

 (P08) … the hospital staff doesn't cooperate (in recording the data) at all, because of their heavy workload, even if they are paid more…

Structural

According to the participants’ experiences, some of main structural challenges are that interventions rely on individual's interest; there is no systemic perspective and no administrative stability. Most of the participants commented that when there is a change of managers in an organization, anything that has been planned stops as well. Another challenge is lack of a systemic approach in road safety management.

 (P04) … this (merging the health and treatment wards of the prevention department in the Ministry of Health) has happened twice in our department. We should be mentioned in the Guinness Book of Records. All of the department and its staff were transferred to another office and the structure completely changed …

Financial

Based on the opinions of participants, financial constraint is a challenge in establishing a road traffic injury surveillance system in Iran. There are limitations as regards the allocation of a sufficient budget for system automation, improvement of infrastructure, advanced technologies and employment of a dedicated registrar.

 (P03) we wanted to update our software but never had the finances needed to do it… (P08) … For the registrar, the money per cases that they receive is important and most of them have financial difficulties…

**Coherence and coordination**

Communication

In this subcategory, the challenges were poor internal and external communication between organizations and the absence of a link between systems. One of the most important pillars of establishing a surveillance system is establishing systematic connections between the stakeholders; however, most of the participants believe that there are fundamental weaknesses in this field. Therefore, each system must have the ability to link with other systems, but one of the challenges is the absence of linking between them. For instance, there is no link between the data of pre-hospital actors and the hospital system.

 (P13) … There is no data link between the police and the emergency departments or even between the emergency organizations and the Forensic Medicine Organization…

**Dispersion**

Numerous and disperse registering systems and parallel activities are some of the challenges of this subcategory. Currently, most of organizations record data in a local and separate manner. Their recorded variables are overlapping each other so, this leads to the design of parallel interventions based on limited goals, and thus time and cost are spent on repeated activities.

 (P07) The police have their own registry and their own objectives; as do, the Forensics Medicine Organization and the Ministry of Health and the Red Crescent… (P11) … Unfortunately, there are separate recording systems…

Cooperation

Poor cooperation, competition between organizations and the absence of Memorandum of Understandings (MOUs) were some of the challenges mentioned by most of participants. Cooperation between stakeholders is necessary for communication between them. To strengthen cooperation, joint agreements must be developed. Another challenge related to cooperation is the sense of competition among stakeholders. Each of stakeholders tries to overplay the importance of their data and underplay the importance of the others’ collected data.

 (P05) … we are not required to cooperate with the other organizations in this field … (P04) a lot of the hospitals didn’t cooperate in recording data…

Consensus

One of the challenges was the lack of unified definitions of a road traffic injury surveillance system. Due to the multiagency nature of road crashes, the terminology should be clearly defined. For instance, in recording the mortality data caused by crashes (dead at the scene of the crash, during transferand in hospital), the deaths should be recorded up to 30 days after the crash. There is no consensus about the definition of “dead on scene” or definition of surveillance system, the responsibility of transferring dead on crash scene, clarifying the role of organizations in a crash scene.

 (P10) definition of the surveillance system is a challenge. If you ask 10 people about the definition of surveillance, you might get 10 different answers. … Our definitions must be similar when we want to establish "injury surveillance”. This (a common language) is very difficult in our country…

**Context**

Socio-cultural

According to the opinions of participants, one of the challenges was poor teamwork. Most of the activities, which require teamwork, do not show good results. Since establishing a surveillance system requires cooperation among the stakeholders, the Socio-cultural context has a great impact. Moreover, each of the stakeholders tries to prove the accuracy of their own data and believes that lack of valid data in this field is the problem of other stakeholders.

 (P06) … our main problem is that we can’t work as teams… (P09) …the real reason that the road safety promotion didn’t reach its main goal is that safety is meaningless to us…

Political

Based on participants, political factors and considering the data as confidential are barriers of distribution of information. Moreover, political authorities always assume that all information can be misused and disrupt the security of the country. Unfortunately, the spread of this viewpoint in the country has made establishing a RTIsurveillance system more difficult.

 (P06) …unfortunately data are not easily accessible in our country. (P01) … another reason (for the lack surveillance system) is the political burden of this matter…

## Discussion

To our best knowledge, this study is the first that was investigated the challenges of establishing a RTI surveillance system in Iran, employing qualitative study. The most important challenges are the absence of a lead agency, lack of a systemic approach to surveillance, failure in stakeholder recognition, unclear objectives, failure in developing a shared plan between stakeholders and lack of ` data dissemination and giving feedback.

One of the main barriers to establishing a RTI surveillance system is the lack of a lead agency. Absence of a specific lead agency leads to failure in stakeholder coordination and cooperation and lack of responsibility for safety promotion among stakeholders. Findings of other studies also imply that the absence of a specific custodianleads to poor policy- making, failure in resource allocation for safety promotion and injury prevention field, ^12^ and even other kinds of leadership.^31^ In the present study, the important point is that due to poor systematic communication between the stakeholders and their weak interaction with each other, there is no consensus about choosing an organization as a lead agency. This is due to the fact that each stakeholder considers itself as the best candidate. Accordingly, most of designed plans and interventions are executed in the related organizations in a parallel manner and consequently, time and finances are misspent. The results of other studies also confirm that the implementation of plans and interventions without custodianship is ineffective due to lack of consistency and execution guarantee. ^1^ So consensus for choosing one of the organizations or creating a council consisting of all of the stakeholders as a lead agency with legislation rights can organize the dispersed and non-coherent measures in recording, interpretation, and dissemination of the data related to crashes.

Lack of a system approach is another barrier which must be dealt with in order to establish an injury surveillance system. This challenge in road safety leads to the design of superficial and ineffective plans and interventions, so that; interventions are designed and executed in order to reduce human errors. This approach is mainly observed in LMICs; whereas in the system approach, which usually is seen in high income-countries, plans and interventions focus on the whole system and there is a division of responsibilities between the stakeholders and the road users. A system approach to the RTIs before and after the crashes is also mentioned as a necessity in other studies conducted in Iran.^32^ In this approach the focus shifts from only paying attention to the human errors toward the identification of the weaknesses in the system. For instance, it seems that by conducting the “Vision Zero” in Iran, a system approach can be established among the authorities and policy makers in road safety. Accordingly, all the interventions and plans will be formulated and executed based on the reduction of fatality and injury of the road users and the main responsibility falls on the system, therefore the focus would be more on the environment rather than reduction of human errors. Also, the findings of other study confirm that applying the Vision Zeroapproach in LMICs can be effective in creating a systematic perspective in the context of road safety.^33^

It is noteworthy that in this study, due to the organizational variation as well as numerous factors contributing to road safety, there is no systematic communication between the stakeholderswhich leads to insufficient knowledge about the objectives and operations of other stakeholders.^25^ According to the WHO guidelines, one of the main steps in establishing an injury surveillance system is recognition of the key stakeholders.14 An important challenge in this step is that the organizations are not willing to share their data with each other and consider their own data as the most complete and important one. The above challenge can be overcome by creating a comprehensive system, consisting of different representatives for road safety.

The first step in surveillance establishment is achieving an understanding of the whole system and its objectives by policy makers and stakeholders.^10^ Since the objective of recording the data in some organizations is not aligned with the objectives of the surveillance system, the data generated by the existing systems is not sufficient for developing safety promotion and injury prevention strategies. In addition, due to lack of understanding of the system and its objectives, definitions and terms are diversified and the stakeholders only focus on the collection of information of RTIs, so interpretation and dissemination of data is neglected by the stakeholders. It also can result in missing some important information particularly related to hot spot analysis and spatial analysis which is emphasized by other related studies,^34^ and its effectiveness in showed in most studies.^35^ It is also important to note that lack of understanding of the system also resulted in the ignoring of the timeliness of the dissemination of RTI information. While efforts were made by various organizations for data collection; however, late dissemination of RTI information affected an important timeliness attribute of surveillance. Focusing on this challenge, there is no reliable estimation of RTIs burden in the study setting. The findings of other studies show that this challenge is mostly observed in LMICs countries.^11,13,36,37^ In order to overcome this challenge, deciding on a lead agency with adequate authority is an important step.

Lack of collaborative plans among the stakeholders is another barrier to the establishment of injury surveillance, because it reduces the effectiveness of road safety interventions which lead to unfavourable results. Due to the dispersion of data in different organizations, the prioritizing of interventions and clarifying the roles of stakeholders in this field is not conducted correctly and therefore the interventions are not carried out with the contribution of all the stakeholders. Moreover, the role of the supervision sector is weak and the designed plans are discontinued half-finished. Having a coherent and integrated data system with the contribution of all the stakeholders and organizations, in a way that each organization is aware of their role, can improve injury surveillance systems in Iran.

Dissemination of data and giving feedback is also another barrier to road traffic injury surveillance in this study. Other studies have stated the reasons for neglecting the dissemination of the data are the result of the lack of trained staff, defective dissemination infrastructures, poor distribution networks, and providing reports that are hard to understand for the beneficiaries.^38-41^ But in this study, the most important reasons for the dissemination failures are that the stakeholders consider only their own personal interests in dissemination or they consider the data as confidential. It seems that neither the importance of data dissemination and giving feedback, nor the need for properly designed infrastructures for data distribution, are understood by the stakeholders. It is important to note that due to a lack of copyright policies in Iran, this problem is encountered in all fields and therefore data is not easily accessible for individuals and organizations. Thus, initially the importance of the dissemination of data must be understood especially through joint workshops and conducting international/ national conferences related to this field between stakeholders.

**The strengths and limitations of the study**

One of the limitations of the study was the difficulties involved in accessing the stakeholders and the bureaucracy involved in scheduling meetings with them. We overcame this limitation by creating interactions. Moreover, compared to quantitative studies, the number of participants in this qualitative study is small. However, the amount of data with in-depth face-to-face interview was quite rich with large amount of data as well as data triangulation that all resulted in more trustworthy information.

## Conclusion

Lack of an RTI surveillance system is one of the major challenges in road safety in Iran. There is an urgent need for a recognition and prioritization of strategies for road safety promotion and injury prevention. This study presents the barriers to the creation of an RTI surveillance system in Iran. We believe that by determining a lead agency for road safety, identifying data collection systems, determining a clear goal for increasing road safety and developing policies for dissemination of data among organizations, it will be feasible to overcome the obstacles to establishing an RTI surveillance system.

**List of Abbreviations**

RTIs: Road traffic injuries; LMICs: Low- and middle-income countries; WHO: World Health Organization; YLL: Years of Life Lost; MOHME: Ministry of Health and Medical Education.

**Acknowledgement**

This paper was part of a PhD thesis in Shahid Beheshti University of Medical Sciences (SBMU), School of Public Health and Safety. The authors would like to thank all participants for their cooperation in this study.
